# Selective synergistic effects of oxalic acid and salicylic acid in enhancing amino acid levels and alleviating lead stress in *Zea mays* L

**DOI:** 10.1080/15592324.2024.2400451

**Published:** 2024-09-05

**Authors:** Minoti Gupta, Swatantar Kumar, Vinay Dwivedi, Dikshat Gopal Gupta, Daoud Ali, Saud Alarifi, Ashish Patel, Virendra Kumar Yadav

**Affiliations:** a Department of Biotechnology, University Institute of Biotechnology, Chandigarh University, Chandigarh, India; b Department of Biotechnology Engineering & Food Technology, University Institute of Engineering, Chandigarh University, Chandigarh, India; c Amity Institute of Biotechnology, Amity University, Gwalior, India; d Department of Urology & Pathology, The Robert H. Lurie Comprehensive Cancer Center, Northwestern University Feinberg School of Medicine, Chicago, IL, USA; e Department of Zoology, College of Science, King Saud University, Riyadh, Saudi Arabia; f Department of Life Sciences, Hemchandracharya North Gujarat University, Patan, India; g Department of Microbiology, Faculty of Sciences, Marwadi University Research Center, Marwadi University, Rajkot, India

**Keywords:** Abiotic stress, stressors, free radicals, malondialdehyde, salicylic acid

## Abstract

Lead is one of the major environmental pollutants which is highly toxic to plants and living beings. The current investigation thoroughly evaluated the synergistic effects of oxalic acid (OA) and salicylic acid (SA) on *Zea mays L*. plants subjected to varying durations (15, 30, 30, and 45 days) of lead (Pb) stress. Besides, the effects of oxalic acid (OA) combined with salicylic acid (SA) for different amino acids at various periods of Pb stress were also investigated on *Zea mays* L. The soil was treated with lead nitrate Pb (NO_3_)_2_ (0.5 mM) to induce Pb stress while the stressed plants were further treated using oxalic acid (25 mg/L), salicylic acid (25 mg/L), and their combination OA + SA (25 mg/L each). Measurements of protein content, malondialdehyde (MDA) levels, guaiacol peroxidase (GPOX) activity, catalase (CAT) activity, GSH content, and Pb concentration in maize leaves were done during this study. MDA levels increased by 71% under Pb stress, while protein content decreased by 56%, GSH content by 35%, and CAT activity by 46%. After treatment with SA, OA, and OA+SA, there was a significant reversal of these damages, with the OA+SA combination showing the highest improvement. Specifically, OA+SA treatment led to a 45% increase in protein content and a 39% reduction in MDA levels compared to Pb treatment alone. Moreover, amino acid concentrations increased by 68% under the Pb+OA+SA treatment, reflecting the most significant recovery (*p* < 0.0001).

## 1. Introduction

Globally, a tremendous increase in heavy metals (HM) contamination has been observed due to rapid industrial growth, anthropogenic activities, and agricultural practices, emphasizing the necessity for monitoring and mitigation measures.^1^ Elevated oxidative stress (OS) from metal ion accumulation in plants due to soil pollution inhibits seed germination, constrains plant growth, and disrupts the absorption of essential minerals, adversely affecting plant development.^2,3^ In recent times, scientists have been delving into the mysteries surrounding HM contamination and its detrimental effects on plants. HM contamination significantly impacts the growth and development of plants, with potential consequences for humans and animals.^4,5^ HMs disrupt the balance within plant cells, known as homeostasis, and also lead to several harmful effects on biological functions like acute poisoning and nucleic acid alterations, which result in elevated levels of reactive oxygen species (ROS).^6,7^ These elevated ROS negatively affect physiological activities like aerobic respiration and photosynthesis in plants.^8,9^ These factors play a pivotal role in influencing the plant’s ability to tolerate and adapt to various environmental and biological stressors.^10^

The toxicity due to HMs toxicity disrupts water and nutrient intake, which affects crucial activities like embryo development, flowering, and seed formation.^11,12^ To overcome the ROS effects, plants develop an antioxidant defense system, which produces enzymes like catalase (CAT), superoxide dismutase (SOD), and the antioxidant enzyme GSH peroxidase (GPOX), they limiting the plant’s ability to tolerate both biotic and abiotic stressors.^13,14^ There are several indicators in plants, such as glutathione, malondialdehyde (MDA), and protein content, which provide insights into OS and the response of the plants to environmental challenges. The absorption of HMs by plants disrupts the balance of ions, prompting plants to induce a detoxification mechanism that involves the formation of metal-binding proteins called phytochelatins.^15^ The activity of the phytochelatin synthase results in the formation of phytochelatins, which act as a chelator to support the HMs detoxification mechanism.^16,17^

Among various HMs, Pb stands out as a noteworthy environmental inorganic pollutant that originates from different sources like emissions from vehicle exhaust, fertilizers, pesticides, and substances added to pigments and gasoline.^18,19^ Moreover, Pb could easily accumulate in the soil and sediments and the presence of the excess amount of Pb has negative impacts on the development and growth of the plant. The Pb accumulation in plants may interfere with the absorption of crucial minerals and may also induce hormonal imbalances and OS.^20^ From the various pieces of literature, it has been found that Pb could significantly impact the transpiration of plants, biosynthesis of chlorophyll, and cellular organelle structure.^21^

The contamination of Pb crops especially in maize challenges a potential global threat as it infiltrates the food chain, which may cause severe health risks.^22^ Human exposure to Pb may cause severe neurotoxicity mainly in children, which may result in cognitive and developmental issues.^23^ Moreover, in a prolonged period, Pb accumulates in the biological systems, which may cause long-term health problems.^24^ WHO organized International Lead Poisoning Prevention Week (ILPPW) to solve the problems arising from the Pb, which mainly emphasizes the critical issue of Pb toxicity.^25^

Various soil factors like pH, clay content, carbon, and oxides influence the absorption of HMs or Pb by plants.^1^ Plants have several defensive mechanisms for the detoxification of HMs or Pb, which is crucial for their survival. The alleviated oxidative damage and increasing resistance to environmental stresses are often associated with an efficient antioxidative system, which comprises both enzymatic and non-enzymatic antioxidants. The chelation of metal ions is a key defense strategy in plants under metal stress. Several biomolecules like proline, polyamines, and non-protein thiols (NPT) like cysteine, glutathione (GSH), and phytochelatins (PCs) are associated with metal chelation in the cytoplasm, which aids in mitigating negative effects (Hall 2002). One such metal chelating agents are oxalic acids (OA) which play a vital role in defense strategy in plants under metal stress.

OA is a naturally occurring metabolite in plants that includes various forms like calcium oxalate crystals, free acid, and soluble salts.^26^ OA has an important role in metal chelation in bacteria, fungi, and plants where metabolic pathways like the tricarboxylic acid (TCA) cycle and glyoxylate pathway play a significant role in the formation of metal complex.^27,28^ In fungi and plants, OA acts as an electron acceptor in the manganese peroxidase catalytic cycle, which regulates oxygen species via the Fenton reaction, which is important for survival. Besides this, OA also acts as a pH and osmoregulator^29^ and due to its low pKa values (1.23 and 4.19), OA facilitates hydrogen electron release. In addition to this, the higher solubility capacity of OA forms complexes with HM ions, which reduces their fixation, mobility, availability, and toxicity to plants.^30^

Another compound is salicylic acid (SA), which is essential for regulating the metabolic process of ROS and protecting plants from diverse stresses. Besides this, SA also acts as a significant signaling molecule for plants, which plays an important role in their response to environmental challenges with dual effects and hormone-like features. When the SA concentration is low then it enhances plant tolerance to various abiotic stimuli. When the SA concentration is high, it can induce OS, which results in the mortality of the plant.^20,31^ Earlier investigation revealed the contribution of SA in maintaining nutrient balance in plants and controlling the intake of Pb. From the various investigations, it has been observed that SA effectively prevents the absorption of Pb, partially mitigating Pb-induced changes in the concentrations of manganese (Mn), calcium (Ca), and iron (Fe) in *Vallisneria natans* leaves exposed to Pb.^32,33^

Zanganeh et al. studied the possible ameliorating effect of SA at the biochemical level in Pb-stressed maize plants. A decrease in the protein thiol level and an increase in the H_2_O_2_ and malondialdehyde contents was noticed under the Pb stress. The study concluded that how salicylic acid helps maize plants cope with Pb stress by reducing oxidative damage, protecting chlorophyll and proteins, and enhancing antioxidant defenses.^34^

One of the investigations carried out by Li et al. exhibited that the treatment of maize seeds with salicylic acid and H_2_O_2_ together enhances seed growth, stress resistance, and chilling tolerance by enhancing antioxidant defenses, hormone levels, and energy supply.^35^

Both the above studies reveal different but complementary insights into the role of SA in mitigating stress in maize plants. Zanganeh et al. 2018, investigated the biochemical effects of SA on maize plants under Pb stress, while Li et al. 2017 observed the combined effects of SA and H_2_O_2_ on maize seed germination, growth and stress resistance under low temperatures. So, following an exhaustive literature survey, no study has yet investigated the combined effects of SA and OA on *Zea mays* plants under Pb stress up to this point.

The research articles aim to investigate the specific protective mechanisms of oxalic acid and salicylic acid alone and in combination on *Zea mays* plants under Pb stress. One of the objectives was to determine the distinct roles of oxalic and salicylic acid in mitigating the adverse effects of Pb contamination. The next objective was to evaluate the antioxidant responses, including malondialdehyde levels, protein content, amino acids content, and glutathione level (GSH), along with the activity of antioxidant enzymes (GPOX and CAT). A further objective was to analyze the Pb content in *Zea mays* leaves, with the overarching goal of enhancing maize yield in HM-contaminated soil by harnessing the protective capabilities of the oxalic acid and salicylic acid combination.

## 2. Materials and methods

### 2.1. Materials

Suvarna Maize (*Zea mays L*.) seeds (Chandigarh, India), oxalic acid, and salicylic acid were procured from Sigma-Aldrich (Sigma Aldrich, St. Louis, MO, USA), double distilled water (ddw) [Thermo fisher scientific, Waltham, MA], lead nitrate [Pb (NO_3_)_2_] [Sigma-Aldrich (Sigma Aldrich, St. Louis, MO, USA].

### 2.2. Experimental set-up

The present study was carried out in the Department of Biotechnology, University Institute of Biotechnology, Chandigarh University, India. A pot experiment was conducted during the summer season of 2021– 2022 to evaluate the impact of Pb on maize crops. A stock solution of OA and SA was prepared i.e., SA (25 mg/L), OA (25 mg/L), and OA + SA (25 mg/L each) in ddw. Lead nitrate was used as a source of Pb for in vivo experiment. The inhibitory concentration (IC-50) for Pb was determined under controlled environmental conditions using a seed germinator. The IC-50 concentration represents the level at which 50% of growth parameters (shoot length, root length, and biomass) were assessed for developing seedlings under controlled conditions (Supplementary Table S1). For experimentation, both a control group (Pb 0 mM) and the IC-50 concentration for Pb (0.50 mM) were selected. The standalone & the combination of Pb, OA, SA, and OA + SA for in vivo studies in *Zea mays*, as shown in Table 1.

**Table 1.** Combinations of Pb and OA, SA, and OA+ SA selected for in vivo studies.

Pb combinations	Dose
Control	0
Pb	0.5 mM
Pb + SA	0.5 mM + 0.18 mM
Pb + OA	0.5 mM + 0.28 mM
Pb + SA + OA	0.5 mM + 0.18 mM + 0.28 mM

### 2.3. Pot preparation and fertilizer application

#### 2.3.1. Soil collection site

**Figure 1.**
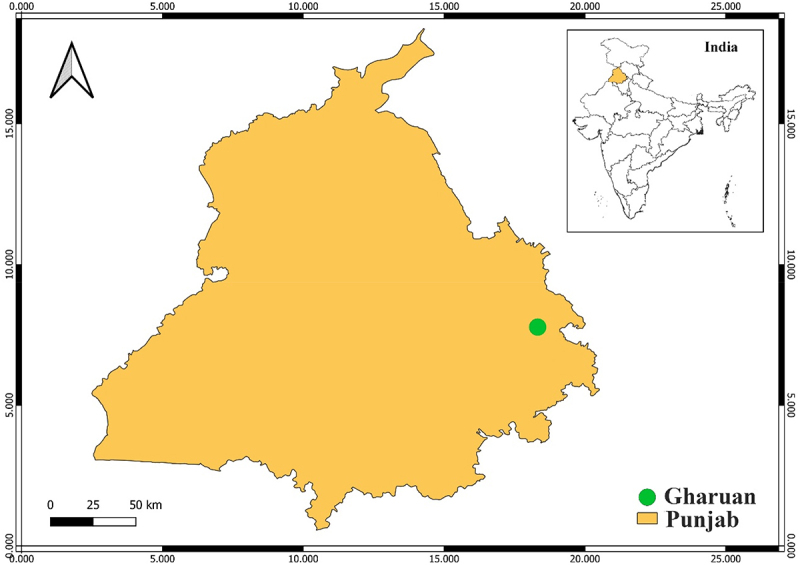
Sampling site of soil collection.

The soil was collected from the Gharuan, Kharar, Mohali district, Punjab, and used in these pot experiments (Figure 1). The soil was sandy loam in texture with a pH of 7.8, organic carbon concentration (%) of 0.81, total nitrogen concentration (%) of 0.119, an available phosphorus (P) amount (mg kg^−1^ soil) of 20.4, exchangeable potassium (K) (meq/100 g soil) amount of 0.294. The salinity of the soil was 0.43 dS/m. The concentrations of Zn in the soil sample were measured to be 9.6 mg/Kg, Fe was 7.19 mg/Kg, Mn was 12.8 mg/Kg, and Cu was 1.32 mg/Kg.

The size of the pot was 15.5 cm (diameter) × 13.5 cm (height). Each pot was filled with 2.228 kg of soil mixed with manure (3:1 ratio). A uniform distance of 9 inches was maintained between each pot.

### 2.4. In vivo cultivation and seed priming for enhanced plant tolerance

For optimizing plant growth and stress resilience, a comprehensive experimental protocol was employed for the in vivo cultivation of *Zea mays*. To ensure seed surface sterility, *Zea mays* seeds surface sterilized by soaking them in 10% sodium hypochlorite solution for 15 minutes, followed by thorough rinsing with distilled water. Subsequently, seed priming, a pivotal pre-germination treatment, was conducted for 12 hours, employing various solutions: distilled water (control), SA at 25 mg/L (0.18 mM), OA at 25 mg/L (0.28 mM), and a combination of SA 25 mg/L (0.18 mM) and OA 25 mg/L (0.28 mM). Post-priming, seeds were germinated on moistened filter paper over 3 days. The experiment incorporated three biological replicates, each comprising five pots representing distinct treatment groups: control (Pb 0 mM), Pb (0.5 mM), Pb + OA (0.5 mM +0.28 mM), Pb + SA (0.5 mM +0.18 mM), and Pb + OA + SA (0.5 mM +0.28 mM +0.18 mM). Each pot accommodated 4 plants, constituting a single experimental replicate, thereby providing a robust foundation for evaluating the effect of priming treatments on subsequent plant responses to the exposure of Pb.

### 2.5. Pb-stress induction and growth conditions for maize plants

To investigate the impact of Pb stress on *Zea mays*, a systematic approach was adopted for the induction of stress and subsequent cultivation. The bifurcation of the maize plants was done into two groups, with one group serving as the control [0 mM of Pb (NO_3_)_2_] and the other subjected to Pb stress [0.5 mM of Pb (NO_3_)_2_]. The growing conditions were accurately controlled, with each replicate consisting of 5 pots containing a soil and manure mixture in a 3:1 ratio. Further, the Pb-treated plants were subjected to a 1-liter solution of Pb (NO_3_)_2_ with a concentration of 0.5 mM applied to the soil before sowing. Whereas the control group pots were irrigated with regular water before seed planting. Following the plantation of seedlings into their respective pots, all plants were positioned outdoors under natural sunlight conditions. The plants were subjected to regular watering for a duration of 15 days, 30 days, and 45 days to maintain optimal growth conditions. After each time interval, maize plants were harvested, and their leaves were precisely preserved at −80°C for subsequent analyses.

### 2.6. Determination of physiological parameters of Zea Mays

The total protein content over here was estimated by using the protein quantification method described by Lowry et al. (1951).^36^ For lipid peroxidation (MDA) quantification, we authors have followed the protocol described by Heath and Packer et al. (1968).^37^ For Pb accumulation, the samples were assessed at 217 nm absorbance using a Perkin-Elmer atomic absorption spectrophotometer (USA). The determined Pb level in samples was expressed as mg g^−1^ dry weight (DW), with replications maintained and computations based on their average. Using a spectrophotometer, the activities of enzyme antioxidants such as peroxidase and catalase were measured. For measuring catalase activity, we used the method described by Aebi et al. (1984).^38^ To calculate the activity of guaiacol peroxidase, we used the method described by Putter et al. (1974).^39^ For the estimation of non-enzymatic antioxidants (GSH content), the authors have used the method described by Sedlak and Lindsay et al. (1968).^40^ For the estimation of amino acids, the protocol of Iriti et al. (2005) was followed.^41^

### 2.7. Statistical analysis

Descriptive data will be presented as median & and range. The normality distribution of data will be determined using the Shapiro – Wilk test/Kolmogorov Smirnov test. Student’s t-test will be used for continuous variables and uniformly distributed data between 2 groups. Mann-Whitney U/Kruskal Wallis will be performed for non-uniformly distributed data. 2-way ANOVA followed by a post hoc Tukey test will be performed to rule the statistical significance. Values of p less than 0.05 will be considered statistically significant and represented *p < 0.05, **p < 0.01, ***p < 0.001, ****p < 0.0001. All statistical tests will be two-tailed, with a significance level of *p* < 0.05. All the statistical analysis will be performed using licensed GraphPad Prism (v9.2) & and SPSS 22.0 (IBM SPSS Inc. NY, US).

## 3. Results

### 3.1. Modulatory effects of oxalic acid and salicylic acid on protein content in Pb-stressed plants

**Figure 2.**
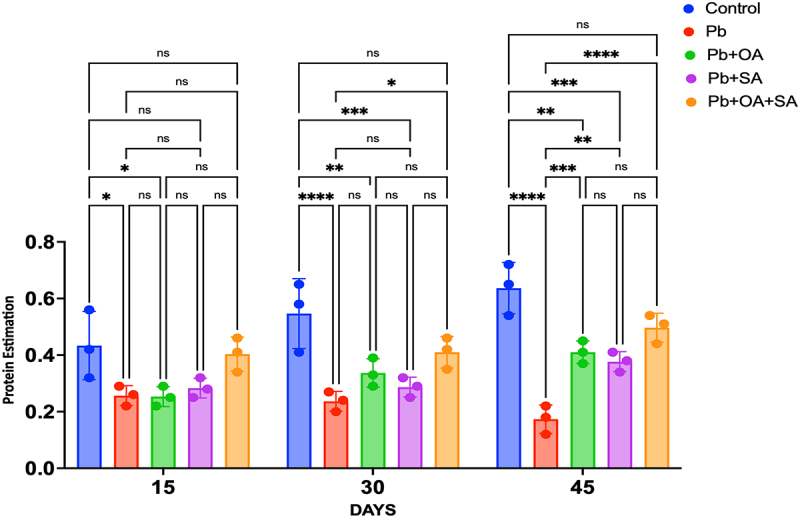
Effect of Pb (0.5 µm) on protein content (µg/l) in maize plants treated with oxalic acid (OA), salicylic acid (SA), and a combination of both (OA+SA) over 15, 30, and 45 days. Each bar represents the mean of three biological replicates, with each replicate consisting of five pots containing four plants. Error bars represent the standard error of the mean (SEM).

Exposure of maize plant leaves to 0.5 mM Pb led to a substantial, time-dependent reduction in protein content: 169.0% at 15 days, 228.0% at 30 days, and 368.1% at 45 days, as detailed in Figure 2. Treatment with OA at a concentration of 0.28 mM demonstrated a substantial augmentation in protein content, with increases of 116.9% at 15 days, 140.4% at 30 days, and 236.9% at 45 days compared to Pb-stressed plants. Similarly, the application of SA at the same concentration resulted in a significant elevation in protein content, showing increments of 110.4% at 15 days, 119.5% at 30 days, and 219.6% at 45 days compared to Pb-stressed plants. Combining OA and SA (25 mg/L (0.28 mM and 0.18 mM) each) produced remarkably significant results, indicating a substantial increase in protein content: 157.3% at 15 days, 191.9% at 30 days (*p* < 0.05), and 287.1% at 45 days (*p* < 0.05), as shown in Figure 2. These findings elucidate the individual and combined efficacy of OA and SA in mitigating the deleterious effects of Pb stress on protein synthesis in plants over varying time intervals.

### 3.2. Synergistic effects of oxalic and salicylic acid on Pb-stressed plants

**Figure 3.**
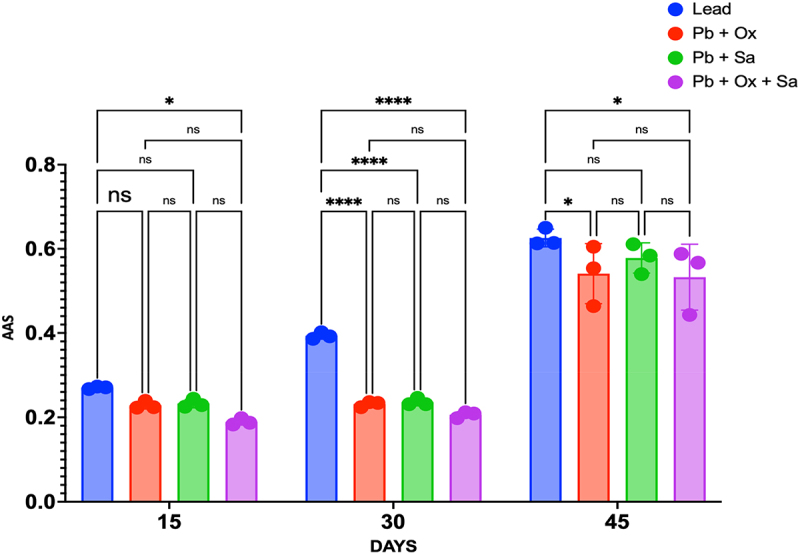
Concentration of Pb (mg/g DW) in maize plants treated with Pb (0.5 mM), Pb + OA (0.5 mM + 25 mg/L (0.28 mM)), Pb + SA (0.5 mM + 25 mg/L ((0.18 mM)), and Pb + OA + SA (0.5 mM + 25 mg/L (0.28 mM) + 25 mg/L (0.18 mM)) over 15, 30, and 45 days. Each bar represents the mean of three biological replicates (*n* = 20), with error bars indicating SEM.

Pb concentration in maize leaf samples, treated with 0.5 mM Pb, exhibited a time-dependent increase: 0.271 mg g^−1^ DW at 15 days, 0.393 mg g^−1^ DW at 30 days, and 0.626 mg g^−1^ DW at 45 days. Treatment with OA at a concentration of 0.28 mM to Pb-stressed plants resulted in a marginal reduction in Pb concentration at 15, 30 & 45 days compared to plants treated with Pb alone. Similarly, treatment with SA at the same concentration exhibited a decline in Pb concentration at 15, 30 & 45 days compared to plants treated with Pb alone. Combining both OA and SA (25 mg/L each) in Pb-stressed plants resulted in a significant reduction of Pb concentration at 15, 30 & 45 days (p < 0.05) compared to plants treated with Pb alone, as shown in Figure 3.

### 3.3. Effect of oxalic and salicylic acids on MDA levels in Pb-stressed plants

**Figure 4.**
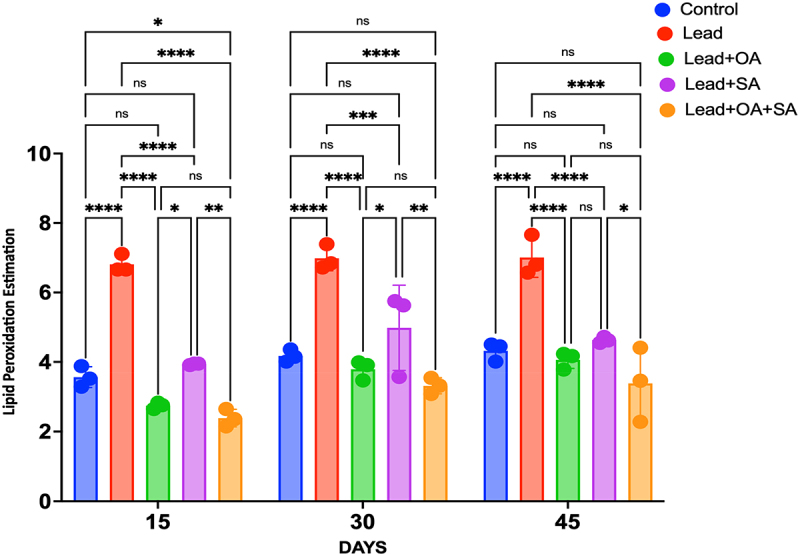
Malondialdehyde (MDA) content (µm/g FW) in maize plants under Pb stress treated with OA, SA, and a combination of both (OA + SA) over 15, 30, and 45 days. The values are based on three biological replicates, with each replicate comprising 20 plants. Error bars represent SEM.

Pb-treated maize plants showed elevated levels of MDA compared to the control group, while treatment with SA, OA, and their combination (OA + SA) effectively mitigated the accumulation of MDA in Pb-treated plants. Here, the authors have assessed the impact of Pb stress on plants and the modulation of MDA content; the MDA levels in control plants exhibit stability at 0.14 µM g^−1^ FW at 15 days, 0.28 µM g^−1^ FW at 30 days, and 0.37 µM g^−1^ FW at 45 days. Conversely, Pb-treated plants display an increasing trend in MDA content over time, registering values of 0.23 µM g^−1^ FW at 15 days, 0.39 µM g^−1^ FW at 30 days, and 0.72 µM g^−1^ FW at 45 days. Treatment with OA to Pb-treated plants results in a marginal decrease in MDA content, ranging from 0.16 µM g^−1^ FW at 15 days to 0.20 µM g^−1^ FW at 45 days, though inconsistently across time points. Similarly, treatment with SA to Pb-treated plants leads to a slight reduction in MDA content, with values ranging from 0.19 µM g^−1^ FW at 15 days to 0.23 µM g^−1^ FW at 45 days. When both OA and SA are combined with Pb treatment, we observed a significant decrease in MDA content is observed, with values of 0.11 µM g^−1^ FW at 15 days, 0.18 µM g^−1^ FW at 30 days, and 0.18 µM g^−1^ FW at 45 days (*p* < 0.05), as shown in Figure 4.

### 3.4. Influence of OA and SA on antioxidant enzyme activity in Pb-stressed plants

**Figure 5.**
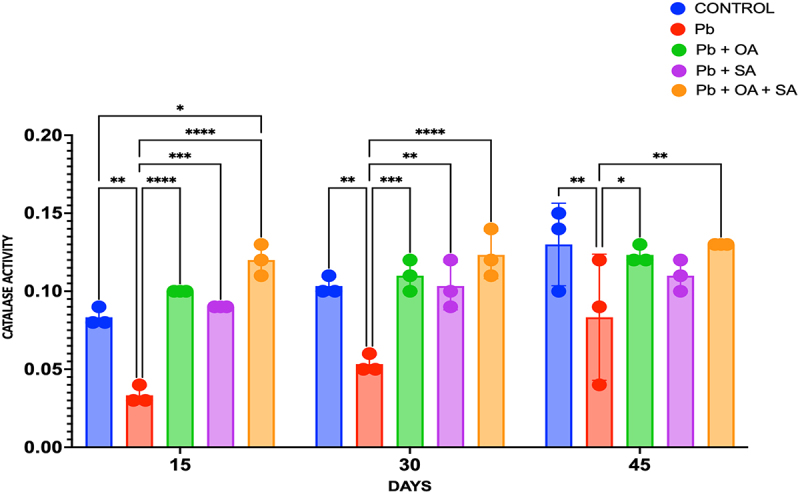
Catalase (CAT) activity (µmol^−1^ g^−1^ protein) in maize leaves under Pb stress treated with OA, SA, and a combination of both (OA + SA) over 15, 30, and 45 days. Each bar represents the mean of three biological replicates (*n* = 20), with error bars showing SEM.

In comparison to control plants unaffected by Pb stress, CAT activity exhibited a decline in Pb-treated plants. However, when subjected to SA, OA, and a combination of both (OA + SA) treatments alongside Pb stress, a significant increase in CAT activities was observed in maize plant leaves (p < 0.05). The specific activity of CAT across distinct time points (15 days, 30 days, and 45 days) was measured in micromoles per gram of protein (µmol^−1^ g^−1^). In the control group, CAT activity at 15 days was 0.08 ± 0.01 µmol^−1^ g^−1^ of protein, which increased to 0.10 ± 0.01 µmol^−1^ g^−1^ at 30 days and further to 0.13 ± 0.025 µmol^−1^ g^−1^ at 45 days. In Pb-treated plants, CAT activity was lower at all time points: 0.03 ± 0.006 µmol^−1^ g^−1^ at 15 days, 0.05 ± 0.01 µmol^−1^ g^−1^ at 30 days, and 0.09 ± 0.04 µmol^−1^ g^−1^ at 45 days compared to the control. Oxalic acid treatment resulted in a small increase in CAT activity, with values of 0.10 ± 0.004 µmol^−1^ g^−1^ at 15 days, 0.11 ± 0.01 µmol^−1^ g^−1^ at 30 days, and 0.12 ± 0.01 µmol^−1^ g^−1^ at 45 days. SA treatment also showed a slight increase, with CAT activity at 0.09 ± 0.004 µmol^−1^ g^−1^ at 15 days, 0.10 ± 0.01 µmol^−1^ g^−1^ at 30 days, and 0.11 ± 0.01 µmol^−1^ g^−1^ at 45 days, although not significantly different from Pb-only treatment. The combination of oxalic acid and salicylic acid significantly/consistently resulted in the highest CAT activity at all time points (p < 0.05): 0.15 ± 0.01 µmol^−1^ g^−1^ at 15 days, 0.16 ± 0.01 µmol^−1^ g^−1^ at 30 days, and 0.20 ± 0.014 µmol^−1^ g^−1^ at 45 days, as shown in Figure 5. In summary, Pb treatment reduced CAT activity, but the addition of OA or SA partially mitigated this reduction. Notably, the combined treatment of Pb, OA, and SA exhibited the highest CAT activity, suggesting a potential synergistic protective effect against Pb-induced CAT inhibition.

**Figure 6.**
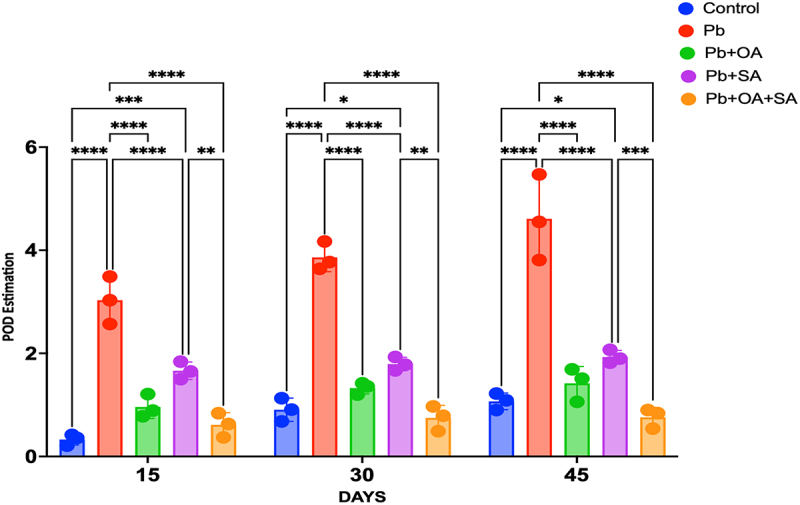
Guaiacol peroxidase (GPOX) activity (µmol^−1^ g^−1^ protein) in maize plants exposed to Pb and treated with OA, SA, and a combination of both (OA + SA) over 15, 30, and 45 days. Error bars represent the SEM of three biological replicates.

In response to Pb stress, there was a significant increase in GPOX activity in maize leaves. However, treatments with SA, OA, and their combination (OA + SA) resulted in a significant reduction of GPX activity in Pb-stressed plants (*p* < 0.05). The impact of Pb on peroxidase activity in control plants remained relatively stable over time, with a gradual increase in Pb-stressed plants, indicating an induction or activation of peroxidase in response to Pb exposure. OA treatment exhibited a suppressive effect on peroxidase activity in Pb-stressed plants, with specific activity values of 0.96 ± 0.22 mol^−1^gm^−1^ of protein (15 days), 1.33 ± 0.11 mol^−1^gm^−1^ of protein (30 Days), and 1.42 ± 0.32 mol^−1^gm^−1^ of protein (45 Days). SA treatment also had an inhibitory effect on peroxidase activity in Pb-stressed plants, with specific activity values of 1.67 ± 0.17 mol^−1^gm^−1^ of protein (15 days), 1.79 ± 0.13 mol^−1^gm^−1^ of protein (30 Days), and 1.93 ± 0.13 mol^−1^gm^−1^ of protein (45 Days). The combination of OA and SA (OA + SA) showed the remarkable lowering of peroxidase activity among all treatments and the control group (*p* < .05), with specific activity values of 0.62 ± 0.24 mol^−1^gm^−1^ of protein (15 Days), 0.75 ± 0.25 mol^−1^gm^−1^ of protein (30 Days), and 0.76 ± 0.19 mol^−1^gm^−1^ of protein (45 Days), as shown in Figure 6. In summary, the guaiacol peroxidase (GPOX) analysis indicates that Pb exposure increases peroxidase activity, while OA, SA, and their combination can modulate this response, either suppressing or stimulating peroxidase.

### 3.5. Investigating the role of OA and SA on non-enzymatic antioxidant activity in Pb-stressed plants

**Figure 7.**
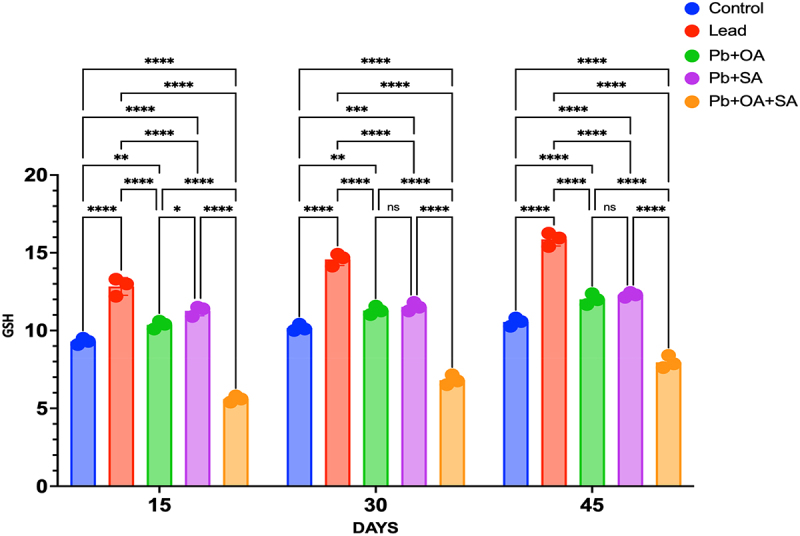
Glutathione (GSH) content (mg/g FW) in maize leaves treated with Pb (0.5 mM), Pb + OA (0.5 mM + 25 mg/L (0.28 mM)), Pb + SA (0.5 mM + 25 mg/L (0.18 mM)), and Pb + OA + SA (0.5 mM + 25 mg/L (0.28 mM) + 25 mg/L (0.18 mM)) over 15, 30, and 45 days. Data are presented as the mean ± SEM of three biological replicates.

Maize plants exposed to Pb stress displayed a significant decrease in GSH content compared to the control group. However, treatments with SA, OA, and the combined OA + SA significantly increased the GSH concentration in Pb-stressed maize leaves (*p* < 0.05). In the control group, without Pb exposure, the GSH content gradually increased over time, reaching 10.55 ± 0.3 mg/gm FW at 45 days. In contrast, the Pb-stressed group consistently exhibited lower GSH content at all time points, with values of 7.96 ± 0.4 mg/gm FW at 45 days. The addition of OA to Pb-stressed plants resulted in a slight increase in GSH content at all time points, reaching 8.45 ± 0.3 mg/gm FW at 45 days. Similarly, SA treatment led to a slight increase, reaching 6.85 ± 0.3 mg/gm FW at 45 days. The combination of OA + SA showed the highest GSH content among all treatments, with values of 8.92 ± 0.2 mg/gm FW at 45 days, and was statistically significant as compared to the control group, as shown in Figure 7. In summary, the control group exhibited an increasing trend in GSH content, while Pb stress consistently led to lower levels. The addition of OA or SA alone, as well as their combination (Pb + OA, Pb + SA, Pb + OA + SA), resulted in varying levels of GSH content, with the combination group (Pb + OA + SA) showing the highest values at all time points.

### 3.6. Investigating the role of OA and SA along with their combination with different amino acids in Pb-stressed plants

Different amino acids have different effects on the plants. This has been observed in Pb-stressed plants by using OA, SA, and combinations of OA+SA and different amino acids. The results of the investigation are provided below and shown in Figure 8.

**Figure 8.**
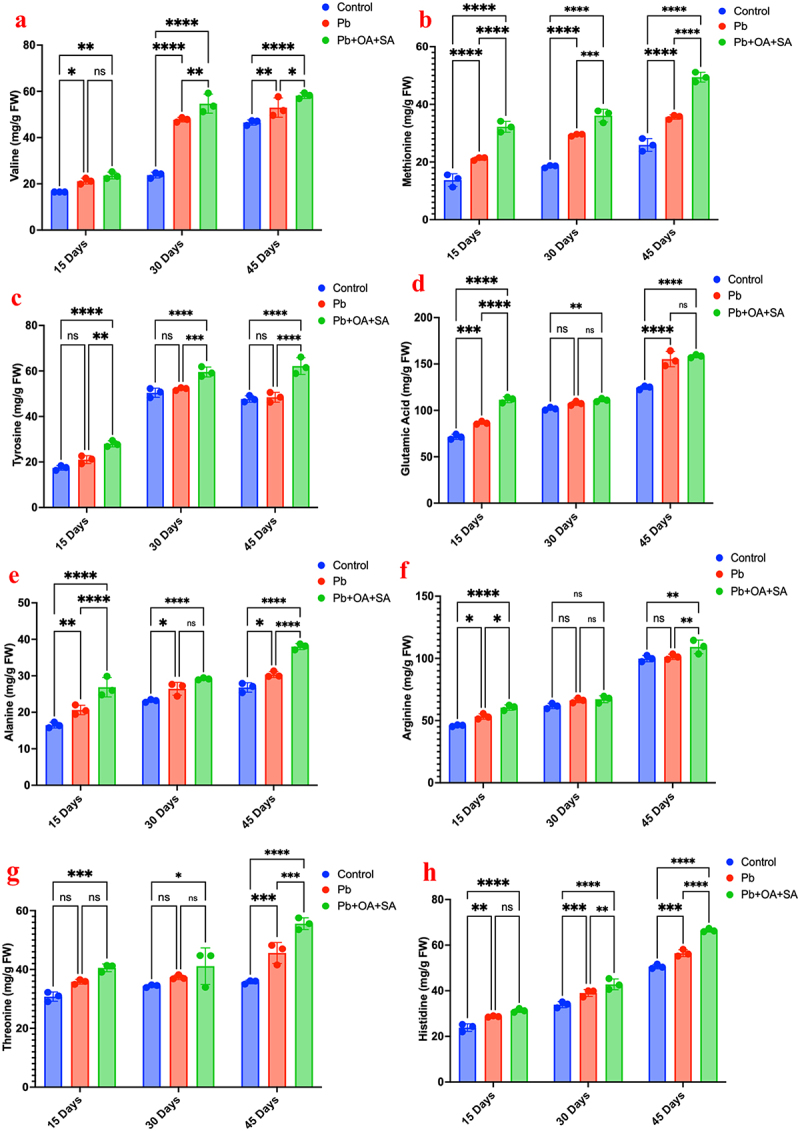
Effect of different amino acids, on various treatments to plants over periods of 15, 30, and 45 days, valine, (b) methionine, (c) tyrosine, (d) glutamic acid, (e) alanine, (f) arginine, (g) threonine, (h) histidine, (i) glycine, (j) serine, (k) proline), (l) lysine, (m) leucine, (n) phenylalanine, and (o) tryptophan.

**Figure 8.**
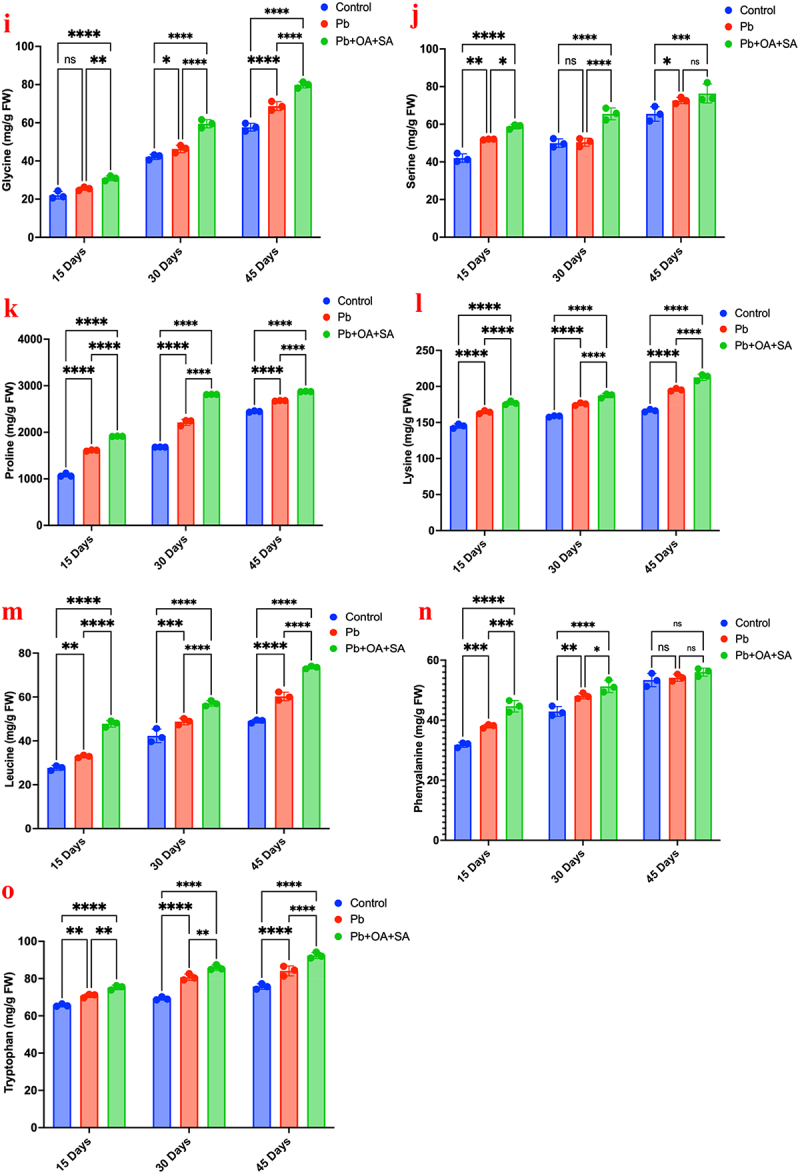
(Continued).

Figure 8 illustrates the various amino acid concentrations (mg/g FW) in plants subjected to three different treatments (control, Pb, and Pb+OA+SA) over periods of 15, 30, and 45 days. At 15 days, a valine concentration was approximately 20 mg/g FW, 20 mg/g FW, and 50 mg/g FW for control, Pb, and Pb+OA+SA treated, respectively (*p* < 0.01). At 30 days, the concentration of valine in the control group remains at about 20 mg/g FW, whereas the significant increases of 40 mg/g FW and 70 mg/g FW for Pb and Pb+OA+SA treatment (*p* < 0.0001). At 45 days, for the control, a steady level of valine 20 mg/g FW was recorded, while for the Pb treatment, a statistically significant rise (*p* < 0.01). Compared to other groups, Pb+OA+SA continues to show the highest concentration, i.e., 70 mg/g FW, significantly surpassing both the control (*p* < 0.01) and Pb treatments (*p* < 0.05). Values of p less than 0.05 will be considered statistically significant and represented *p < 0.05, **p < 0.01, ***p < 0.001, ****p < 0.0001. All statistical tests will be two-tailed, with a significance level of *p* < 0.05.

Methionine levels remain steady in control (~10 mg/g FW); Pb treatment significantly increases methionine, highest at ~30 mg/g FW by 30 and 45 days (*p* < 0.0001). Pb+OA+SA treatment constantly results in the highest methionine levels (~50 mg/g FW) across all time points. Tyrosine remains stable in the control (~20 mg/g FW). Pb treatment shows a non-significant increase initially but reaches (~40 mg/g FW) by 30 and 45 days (*p* < 0.0001). Pb+OA+SA treatment significantly increases tyrosine to (~60-70 mg/g FW) across all time points (*p* < 0.0001). Glutamic acid shows a significant difference between control, Pb, and Pb+OA+SA treatment at 15 days (*p* < 0.0001). The difference is less noticeable at 30 and 45 days. Alanine shows Pb and Pb+OA+SA treatment significantly increases the measured outcome compared to the control across all time points (*p* < 0.01), with Pb+OA+SA having the most pronounced effect. Arginine shows significant differences at 15 days (*p* < 0.05). No significant differences are observed at 30 days. By 45 days, Pb+OA+SA treatment shows a significant increase compared to the control and Pb alone (*p* < 0.01). Threonine shows no significant differences at 15 days. A significant increase is observed at 30 days for control, also for Pb+OA+SA (*p* < 0.05), and at 45 days for all comparisons (*p* < 0.0001).

Tukey’s multiple comparisons test shows significant differences across various treatments and time points. Pb+OA+SA consistently shows the most significant impact across all measured outcomes (*p* < 0.0001), particularly at 30 and 45 days. Overall, Pb treatment increases the levels of various amino acids and also consistently shows the most significant and sustained increases across all time points

## 4. Discussion

The issue of Pb contamination has garnered substantial attention due to its rapid absorption by plants, entering the food chain and adversely affecting both animals and humans. Our findings reveal a notable increase in Pb accumulation within the leaves of maize plants under Pb stress conditions over a span of 15, 30, and 45 days compared to the control group. However, when treated with OA and SA for the same duration, a significant reduction in Pb accumulation in maize plant leaves was observed (*p* < 0.05). This suggests that both OA and SA demonstrate a promising role in alleviating Pb toxicity by hindering the uptake and mobilization of Pb in plants. Kohli et al. also obtained similar results where a decline in the uptake of Pb was observed in *Brassica juncea* plants after the application of SA externally in plant.^42^

The combined use of gamma-aminobutyric acid (GABA) and salicylic acid demonstrated a significant role in mitigating Pb-induced stress through a multifaceted regulatory mechanism involving phytohormones. Studies indicate that phytohormones such as salicylic acid are crucial in modulating ROS levels and enhancing antioxidant responses in plants under heavy metal stress. Recent investigations have revealed that these hormones act as signaling molecules that regulate the antioxidant enzyme activity, which is crucial in scavenging ROS generated during bioremediation processes.^43,44^ Specifically, salicylic acid influences glutathione levels, thereby modulating oxidative stress and potentially improving Pb uptake and tolerance in plants.^43^ The study by Lv et al. (2023) particularly highlights the integration of phytohormones with ROS management pathways, offering enhanced resistance against heavy metal toxicity. Another study underscores the role of phytohormones in regulating oxidative stress responses, further emphasizing their potential to improve bioremediation efficiency.^44^ Moreover, SA plays a role in the plant’s defense against HM toxicity by obstructing the movement of HM ions across the plasma membrane.^45,46^ Similarly, OA treatment lowered the uptake of Cd in stressed *Cicer arietinum* L., attributed to OA’s strong affinity for metallic ions, where it forms complexes and reduces their soil availability.^47,48^ Organic acids, including OA, released by plants under stress enhance tolerance by reducing HM mobility through sequestration into vacuoles.^47,49^ In the case of Pb stress, combining OA and SA at 25 mg/L each over 15, 30, and 45 days significantly decreased the accumulation of Pb in the leaves of maize plants. This points to a synergistic effect,^50^ with SA promoting proline biosynthesis, which chelates Pb, resulting in cytoplasmic sequestration. Simultaneously, OA decreases the uptake of Pb by chelating and sequestering Pb ions, reducing their availability in soil and subsequent absorption by maize plants. The combination proves more effective in alleviating Pb-mediated OS than individual treatments.^45^

The increase in MDA production under stress conditions is attributed to the overproduction of ROS, which may act as an indicator of OS in plants.^51^ MDA is the by-product of lipid peroxidation of the cell membranes of plant cells due to the production of ROS under Pb toxicity.^52^ In this study, maize leaves treated with 0.5 mM of Pb showed higher MDA concentrations compared to the control, with MDA content gradually increasing over 15, 30, and 45 days of Pb treatment. Treatment with OA significantly reduced the MDA content in Pb-stressed maize plants over the 15, 30, and 45 days. This emphasizes OA’s role as a chelating agent that reduces Pb availability in the soil, subsequently lowering Pb uptake and OS.^53^

Likewise, the application of SA to Pb-stressed plants over periods of 15, 30, and 45 days resulted in a decrease in MDA content, which indicated the ability of SA to mitigate OS by reducing the uptake and mobility of Pb ions within plant cells.^54,55^ The above results align with the studies by Alamer et al.. (2020), which highlighted the reduction of MDA content in SA-treated Pb-stressed wheat plants due to increased antioxidant enzyme activities which inhibited the overproduction of ROS.^20^ In our study, when Pb-stressed maize plants were treated with the combination of OA + SA then there was a significant reduction in MDA content when treated individually with OA or SA. The synergistic effect of OA + SA is attributed to OA behaving as an HM ion chelator, which reduces the availability of metal ions in the soil and subsequent plant uptake.^56^ Concurrently, SA functions as a signaling molecule, which activates antioxidant enzymes to restrict the overproduction of ROS. The combined OA + SA treatment exhibited the most significant reduction in the accumulation of Pb in plant cells, emphasizing its efficacy in mitigating OS and limiting MDA accumulation.^57^

In the current study, exposure to Pb resulted in reduced protein content in maize plants over a period of 15 days, 30 days, and 45 days in comparison to the control group. This decrease could be due to the induction of OS, which causes damage to cell membranes and functional damage to proteins.^58,59^ However, when Pb-stressed plants were treated with OA and SA, then the protein levels demonstrated improvement in all treatments i.e., 15 days, 30 days, and 45 days. OA and SA effectively alleviated OS through chelation, sequestration, and compartmentalization of Pb ions. This reduced the availability of Pb ions to plant cells, consequently increasing protein content. SA, acting as a signaling molecule, intervened at the transcription/translation level, ROS, and mitigating OS.^45^ Notably, the combined application of OA + SA to Pb-stressed plants resulted in higher protein content in maize leaves in comparison to plants treated with Pb alone, Pb + OA, and Pb + SA. This suggests that the synergistic effect of OA + SA was more capable of reducing OS, which helps the normal functioning of plants. Similar studies have shown that the accumulation of Pb results in a decrease in plant protein content.^58,59^ Utilizing organic substances like guaiacol, guaiacol peroxidase (GPOX) serves as a regulator of H_2_O_2_ levels within cells.^60^ The specific activity of guaiacol peroxidase (GPOX) in Pb-treated plants was consistently higher than in the control group at 15 days, 30 days, and 45 days. This indicates that GPOX is induced or activated in response to exposure to the Pb, which acts as a scavenger for free oxygen radicals. This finding is supported by reports of increased peroxidase activity under stressed conditions in maize plants treated with Ni. However, contrasting results were observed under excessive Pb concentrations.^61–63^

When the OA was applied to the Pb-stressed plants, the specific activity of peroxidase was reduced in comparison to the Pb-treated plants alone. This suggested that OA may have a suppressive effect on peroxidase activity, indicating reduced OS in Pb-stressed plants.^64^ The application of SA to Pb-stressed plants resulted in slightly lower peroxidase activity in comparison to the Pb-treated group, indicating reduced OS due to decreased availability of Pb ions to plant cells. Similar results were also obtained by Alamer and Fayez (2020), where restored guaiacol peroxidase (GPOX) levels in Pb-stressed parsley leaves treated with SA.^20^

When the synergistic effect of OA and SA was applied to Pb-stressed plants, then the specific activity of peroxidase was reduced among all treatments (Pb + OA, Pb + SA). This indicated that the combined effect of OA and SA effectively decreases the OS in Pb-stressed plants, suppressing the activity of peroxidase.^57^

The results obtained from our study indicated that exposure to Pb has a suppressive effect on CAT activity in treated plants. The obtained results were in agreement with findings from Kaur et al., (2015), who reported reduced CAT activity in plants when exposed to HMs. This reduction in CAT activity could be due to the inhibition of CAT synthesis or enzyme denaturation caused by the accumulation of Pb ions.^65^ However, the addition of OA and SA is likely to mitigate the negative effects of Pb on CAT activity. So, SA involved in stress signaling pathways has been reported to enhance antioxidant defense systems in plants, including the upregulation of CAT activity.^66,67^ OA, with a strong affinity for metal ions, acts as a metal chelator, which decreases the Pb ion’s availability in the soil, thereby lowering the OS in plants and improving CAT activity.^64^ The combined treatment of Pb with both OA and SA resulted in the highest CAT activity among all treatments, which indicated a synergistic effect. The findings were in agreement with the study, which exhibited that the combined application of organic amendments and SA increased the enzymatic antioxidant system in plants under HMs stress.^68^ The synergistic effect of OA and SA may involve stimulating CAT gene expression and increasing the generation of antioxidant compounds, leading to improved CAT activity.

Glutathione (GSH) plays an important role in maintaining a stable redox state by removing excess ROS from various cellular compartments and detoxifying the cell.^69^ The plants treated with Pb for 15, 30, and 45 days, resulted in a decreased GSH content. However, the treatment with OA, SA, and a combination of both enhanced the content of GSH contents in maize plant leaves under Pb stress. In the case of control plants (not exposed to Pb or any additional substances), there was a gradual increase in GSH content over time. This was consistent with studies demonstrating the accumulation of GSH during normal growth and development.^52^ The rise from 9.32 ± 0.2 mg/gm FW at 15 days to 10.55 ± 0.3 mg/gm FW at 45 days highlights its significance in cellular processes and defense mechanisms. Contrastingly, the Pb-stressed group consistently showed lower content of GSH in comparison to the control group at all time points. The obtained results were in agreement with the previous research, which indicated that Pb exposure may disrupt the antioxidant defense system, leading to reduced GSH levels.^70^

When OA or SA was added to Pb-stressed plants, there was a slight increase in GSH content in comparison to the Pb group, which suggested a positive influence on GSH metabolism and/or synthesis. The obtained results were supported by the previous investigations where an increase in the GSH contents was noticed in Ni-stressed maize plants exposed to OA. A team led by Sakouhi et al. (2022) obtained the ameliorative effect of OA on Cd-treated *Cicer arietinum* germinating seeds. Hayat et al. demonstrated the protective role of SA against OS, which can enhance antioxidant enzyme activity.^47,57,71^ When both OA and SA were added to Pb-stressed plants, there was a significant increase in the GSH content in comparison to the plants treated with Pb alone. This suggested a synergistic effect in promoting GSH accumulation and enhancing the plant’s defense system by upregulating GSH synthesis pathways or modulating enzyme activity in GSH metabolism.

Under Pb stress, *Zea mays* demonstrated increased valine levels, notably rising when OA and SA were added, with the highest concentration at 70 mg/g FW, surpassing control levels significantly. The level of tyrosine in the study remained stable for the control and Pb treatments but significantly increased with the treatment of Pb+OA+SA treatment at 15, 30, and 45 days, exhibiting the highest levels consistently. The glutamic acid level in plants shows significant differences between control, Pb-exposed, and Pb with organic acids at different durations, which indicated the impact of Pb exposure on this outcome. Other amino acids (Figure 8e–o), including arginine, threonine, proline, and others, demonstrated varying levels of significance across different time durations. The Pb+OA+SA treatment generally shows higher levels compared to the control and Pb-only treatments, indicating a potential synergistic ameliorative effect. The statistical analysis, performed using Tukey’s multiple comparisons test, revealed significant differences between the groups at various time points for all measured amino acids. These differences are most pronounced at 15 and 30 days, with a slight reduction by 45 days, although Pb+OA+SA treatment continues to demonstrate notable effects. While the combined treatment of OA and SA appears to enhance the mitigation of Pb stress in *Zea mays*, as reflected by higher amino acid concentrations, this effect should be interpreted with caution and not overstated, as the differences, although statistically significant, may not imply a substantially greater biological impact. The data suggests that OA and SA together may provide some additional benefit in reducing Pb stress compared to either treatment alone.

## 5. Conclusion

In the present study, the impact of Pb treatments on maize plants revealed significant OS as indicated by various biochemical changes. Pb toxicity resulted in reduced protein content, GSH levels, and catalase activity, alongside increased MDA content, guaiacol peroxidase (GPOX) activity, and Pb accumulation, highlighting its detrimental effects on maize physiology. The elevated GPOX activity suggested a potential protective response to Pb toxicity. The combined use of oxalic acid and salicylic acid significantly mitigated Pb-induced stress, as evidenced by a 39% reduction in MDA content, a 42% increase in CAT activity, and a 52% reduction in Pb accumulation in leaves. The synergistic treatment also led to a 68% increase in amino acid concentrations, highlighting its potential to enhance maize tolerance to Pb toxicity. These results underline the novelty and effectiveness of the OA+SA combination, presenting a promising strategy for improving plant resilience in heavy metal-contaminated environments. Future research should emphasize the Pb deposition mechanism within the plant, the effects of Pb-polluted air, and the roles of antioxidant enzymes and compounds in free radical scavenging. These investigations will excavate the understanding of Pb stress in maize and help in developing targeted strategies to improve resilience against oxidative imbalance. Moreover, the synergistic effect of combined Pb+OA+SA treatments on amino acid concentration emphasizes the potential of these compounds in increasing the maize tolerance to Pb-induced stress.

## 6. Limitation and future research

The study demonstrates the potential of oxalic acid (OA) and salicylic acid (SA) in mitigating lead (Pb) toxicity in *Zea mays* through selective synergistic effects, several limitations should be acknowledged. First, the observed synergistic effects are not uniformly significant across all measured physiological and biochemical parameters, indicating that the combined treatment may only benefit specific processes or pathways, such as certain amino acid levels and glutathione content. This suggests that the interaction between OA and SA might be more complex than initially hypothesized, requiring further investigation to fully understand the conditions under which this synergy is most effective.

Second, the study was conducted under controlled conditions with specific concentrations and application methods. The results might vary under different environmental conditions, soil types, or with different concentrations of OA and SA. Additionally, the study focused primarily on short-term responses to Pb stress, and the long-term effects of combined OA and SA treatments on plant growth and yield were not fully explored.

Finally, the study did not fully elucidate the molecular mechanisms underlying the selective synergistic effects, leaving open questions about the precise roles of OA and SA in plant defense responses. Further research incorporating molecular analyses and a broader range of stress markers would be valuable to provide a more comprehensive understanding of the observed phenomena.

## Supplementary Material

Supplementary Table S1.xlsx

## Data Availability

“The datasets generated during and/or analyzed during the current study are available from the corresponding author on reasonable request.”

## Abbreviations

GPOX: Guaiacol peroxidase
BSA: Bovine Serum Albumin
DW: Dry Weight
H_2_O_2_: Hydrogen Peroxide
ROS: Reactive oxygen species
SA: Salicylic acid
OA: Oxalic acid
OA + SA: Oxalic acid + Salicylic Acid
CAT: Catalase
POD: Peroxidase
MDA: Malondialdehyde
Pb: Lead
GSH: Glutathione Stimulating Hormone
FW: Fresh weight
TBARS: Thiobarbituric acid reactive substance

## References

[1] Li P, Liu C, Luo Y, Shi H, Li Q, PinChu C, Li X, Yang J, Fan W. Oxalate in plants: metabolism, function, regulation, and application. J Agric Food Chem [Internet]. 2022;70(51):16037–15. doi:10.1021/acs.jafc.2c04787. 36511327

[2] Khan MIR, Fatma M, Per TS, Anjum NA, Khan NA. Salicylic acid-induced abiotic stress tolerance and underlying mechanisms in plants. Front Plant Sci. 2015;6:6. doi:10.3389/fpls.2015.00462. 26175738 PMC4485163

[3] Mehdinia S, Nassehinia H. Environment and water engineering health and environmental effects of heavy metals (cd, Pb, As). Environ Water Eng [Internet]. 2022;8:538–550. www.jewe.ir.

[4] Mittler R. Oxidative stress, antioxidants and stress tolerance. Trends Plant Sci [Internet]. 2002;7(9):405–410. https://www.sciencedirect.com/science/article/pii/S1360138502023129. 12234732 10.1016/s1360-1385(02)02312-9

[5] Mittler R. Abiotic stress, the field environment and stress combination. Trends Plant Sci [Internet]. 2006;11(1):15–19. https://www.sciencedirect.com/science/article/pii/S1360138505002918. 16359910 10.1016/j.tplants.2005.11.002

[6] Ali H, Khan E, Ilahi I. Environmental chemistry and ecotoxicology of hazardous heavy metals: environmental persistence, toxicity, and bioaccumulation. J Chem [Internet]. 2019;2019:1–14. doi:10.1155/2019/6730305.

[7] Núñez SS, Moltó J, Conesa JA, Fullana A. Heavy metals, PAHs and POPs in recycled polyethylene samples of agricultural, post-commercial, post-industrial and post-consumer origin. Waste Manag [Internet]. 2022;144:113–121. https://www.sciencedirect.com/science/article/pii/S0956053X22001519. 35344786 10.1016/j.wasman.2022.03.016

[8] Kärkönen A, Kuchitsu K. Reactive oxygen species in cell wall metabolism and development in plants. Phytochemistry [Internet]. 2015;112:22–32. https://www.sciencedirect.com/science/article/pii/S0031942214003975. 25446232 10.1016/j.phytochem.2014.09.016

[9] Huang H, Ullah F, Zhou D-X, Yi M, Zhao Y. Mechanisms of ROS regulation of plant development and stress responses. Front Plant Sci. 2019;10:10. doi:10.3389/fpls.2019.00800. 31293607 PMC6603150

[10] Cheng S. Heavy metal pollution in China: origin, pattern and control. Environ Sci Pollut Res Int [Internet]. 2003;10(3):192–198. https://eurekamag.com/research/003/792/003792955.php. 12846382 10.1065/espr2002.11.141.1

[11] Hama Aziz KH, Mustafa FS, Omer KM, Hama S, Hamarawf RF, Rahman KO. Heavy metal pollution in the aquatic environment: efficient and low-cost removal approaches to eliminate their toxicity: a review. RSC Adv. 2023;13(26):17595–17610. doi:10.1039/D3RA00723E. 37312989 PMC10258679

[12] Sharma M, Kant R, Sharma AK, Sharma AK. Exploring the impact of heavy metals toxicity in the aquatic ecosystem. Int J Energy Water Resour. 2024; doi:10.1007/s42108-024-00284-1.

[13] Mansoor S, Wani OA, Lone JK, Manhas S, Kour N, Alam P, Ahmad A, Ahmad P. Reactive oxygen species in plants: from source to sink. Antioxidants. 2022;11(2):225. doi:10.3390/antiox11020225. 35204108 PMC8868209

[14] Du K, Huang J, Wang W, Zeng Y, Li X, Zhao F. Monitoring low-temperature stress in Winter wheat using TROPOMI solar-induced chlorophyll fluorescence. IEEE Trans Geosci Remote Sens. 2024;62:1–11. doi:10.1109/TGRS.2024.3351141.

[15] Pirzadah TB, Malik B, Tahir I, Hakeem KR, Alharby HF, Rehman RU. Lead toxicity alters the antioxidant defense machinery and modulate the biomarkers in Tartary buckwheat plants. Int Biodeterior Biodegrad [Internet]. 2020;151:104992. https://www.sciencedirect.com/science/article/pii/S0964830520302110.

[16] Rodrigo MAM, Anjum NA, Heger Z, Zitka O, Vojtech A, Pereira E, Kizek R. Role of phytochelatins in redox caused stress in plants and animals [internet]. In: Shanker A Shanker C. editors. Abiotic and biotic stress in plants. Rijeka: IntechOpen; 2016. p. Ch. 17. doi:10.5772/62160.

[17] Faizan M, Alam P, Hussain A, Karabulut F, Tonny SH, Cheng SH, Yusuf M, Adil MF, Sehar S, Alomrani SO, et al. Phytochelatins: key regulator against heavy metal toxicity in plants. Plant Stress. 2024;11:100355. doi:10.1016/j.stress.2024.100355.

[18] Mishra S, Jaiswal B, Agrawal SB, Agrawal M. Ecological and health risk assessment of different land uses along with seasonal variation in toxic metal contamination around Varanasi city situated in Indo-Gangetic plain. Environ Geochem Health [Internet]. 2023;45(6):3293–3315. 10.1007/s10653-022-01417-3. 36282409

[19] Sidhu GPS, Singh HP, Batish DR, Kohli RK. Alterations in photosynthetic pigments, protein, and carbohydrate metabolism in a wild plant Coronopus didymus L. (Brassicaceae) under lead stress. Acta Physiol Plant [Internet]. 2017;39(8):176. doi:10.1007/s11738-017-2476-8.

[20] Alamer KH, Fayez KA. Impact of salicylic acid on the growth and physiological activities of parsley plants under lead toxicity. Physiol Mol Biol Plants [Internet]. 2020;26(7):1361–1373. doi:10.1007/s12298-020-00830-1. 32647454 PMC7326881

[21] Gupta M, Dwivedi V, Kumar S, Patel A, Niazi P, Yadav VK. Lead toxicity in plants: mechanistic insights into toxicity, physiological responses of plants and mitigation strategies. Plant Signal Behav. 2024;19(1). doi:10.1080/15592324.2024.2365576. PMC11195469 38899525

[22] Kumar S, Islam R, Akash PB, Khan MHR, Proshad R, Karmoker J, MacFarlane GR. Lead (Pb) contamination in agricultural products and human health risk assessment Bangladesh. Water Air Soil Pollut. 2022;233(7):257. doi:10.1007/s11270-022-05711-9.

[23] Sanders T, Liu Y, Buchner V, Tchounwou PB. Neurotoxic effects and biomarkers of lead exposure: a review. Rev Environ Health [Internet]. 2009;24(1):15–46. doi:10.1515/REVEH.2009.24.1.15. 19476290 PMC2858639

[24] Newman N. Lead poisoning. In: Kamat D, Frei-Jones M, editors. Benign hematologic disorders in children. Cham: Springer International Publishing; 2021. p. 31–50. doi:10.1007/978-3-030-49980-8_3 .

[25] WHO. International lead poisoning prevention week. 2023.

[26] Kumar S, Panwar P, Sehrawat N, Upadhyay SK, Sharma AK, Singh M, Yadav M. Oxalic acid: recent developments for cost-effective microbial production. Phys Sci Rev. 2023;9(2):891–907. doi:10.1515/psr-2022-0167.

[27] Grąz M. Role of oxalic acid in fungal and bacterial metabolism and its biotechnological potential. World J Microbiol Biotechnol. 2024;40(6):178. doi:10.1007/s11274-024-03973-5. 38662173 PMC11045627

[28] Palmieri F, Estoppey A, House GL, Lohberger A, Bindschedler S, Chain PSG, Junier P. Oxalic acid, a molecule at the crossroads of bacterial-fungal interactions. 2019; 49–77. 10.1016/bs.aambs.2018.10.001 30798804

[29] Yu G, Ma J, Jiang P, Li J, Gao J, Qiao S, Zhao Z. The mechanism of plant resistance to heavy metal. IOP Conference Series: Earth and Environmental Science; Vol. 310; 14–16 June 2019; Guiyang, China. Institute of Physics Publishing; 2019. doi:10.1088/1755-1315/310/5/052004.

[30] Bali AS, Sidhu GPS, Kumar V. Root exudates ameliorate cadmium tolerance in plants: a review. Environ Chem Lett [Internet]. 2020;18(4):1243–1275. doi:10.1007/s10311-020-01012-x.

[31] Di Palma L, Petrucci E, Pietrangeli B. Environmental effects of using chelating agents in polluted sediment remediation. Bull Environ Contam Toxicol [Internet]. 2015;94(3):340–344. doi:10.1007/s00128-014-1437-9. 25476737

[32] Yüzbaşıoğlu E, Dalyan E. Salicylic acid alleviates thiram toxicity by modulating antioxidant enzyme capacity and pesticide detoxification systems in the tomato (Solanum lycopersicum Mill.). Plant Physiol Biochem [Internet]. 2019;135:322–330. https://www.sciencedirect.com/science/article/pii/S0981942818305709. 30599309 10.1016/j.plaphy.2018.12.023

[33] Yan Y, Pan C, Du Y, Li D, Liu W. Exogenous salicylic acid regulates reactive oxygen species metabolism and ascorbate–glutathione cycle in Nitraria tangutorum Bobr. under salinity stress. Physiol Mol Biol Plants. 2018;24(4):577–589. doi:10.1007/s12298-018-0540-5. 30042614 PMC6041230

[34] Zanganeh R, Jamei R, Rahmani F. Modulation of growth and oxidative stress by seed priming with salicylic acid in *Zea mays* L. under lead stress. J Plant Interact. 2019;14(1):369–375. doi:10.1080/17429145.2019.1629032.

[35] Li Z, Xu J, Gao Y, Wang C, Guo G, Luo Y, Huang Y, Hu W, Sheteiwy MS, Guan Y. et al. The synergistic priming effect of exogenous salicylic acid and H2O2 on chilling tolerance enhancement during maize (*Zea mays* L.) seed germination. Front Plant Sci. 2017;8. doi:10.3389/fpls.2017.01153. PMC5496956 28725229

[36] Lowry OH, Rosebrough NJ, Farr AL, Randall RJ. Protein measurement with the Folin phenol reagent. J Biol Chem. 1951;193(1):265–275. doi:10.1016/S0021-9258(19)52451-6. 14907713

[37] Heath RL, Packer L. Photoperoxidation in isolated chloroplasts. Arch Biochem Biophys. 1968;125(1):189–198. doi:10.1016/0003-9861(68)90654-1. 5655425

[38] Aebi H. Catalase in vitro. Methods Enzymol. 1984;105:121–126. doi:10.1016/s0076-6879(84)05016-3. 6727660

[39] Pütter J. Peroxidases. In: Bergmeyer H-U, editor. Methods of enzymatic analysis. Verlag Chemie Weinhan Germany: Elsevier; 1974. p. 685–690.

[40] Sedlak J, Lindsay RH. Estimation of total, protein-bound, and nonprotein sulfhydryl groups in tissue with Ellman’s reagent. Anal Biochem [Internet]. 1968;25:192–205. https://www.sciencedirect.com/science/article/pii/0003269768900924. 4973948 10.1016/0003-2697(68)90092-4

[41] Iriti M, Rossoni M, Borgo M, Ferrara L, Faoro F. Induction of resistance to gray mold with benzothiadiazole modifies amino acid profile and increases proanthocyanidins in grape: primary versus secondary metabolism. J Agric Food Chem. 2005;53(23):9133–9139. doi:10.1021/jf050853g. 16277413

[42] Kohli SK, Handa N, Sharma A, Gautam V, Arora S, Bhardwaj R, Alyemeni MN, Wijaya L, Ahmad P. Combined effect of 24-epibrassinolide and salicylic acid mitigates lead (Pb) toxicity by modulating various metabolites in Brassica juncea L. seedlings. Protoplasma [Internet]. 2018;255(1):11–24. 10.1007/s00709-017-1124-x. 28573335

[43] Lv Y, Zhao Y, He Y, Wang J, Zheng Y, Chen X, Huang F, Liu J, Yu L. Synergistic effects of gamma-aminobutyric acid and melatonin on seed germination and cadmium tolerance in tomato. Plant Signal Behav. 2023;18(1):18. doi:10.1080/15592324.2023.2216001. PMC10259350 37302802

[44] Zhao Y, Wang Q, Gu D, Huang F, Liu J, Yu L, Yu X. Melatonin, a phytohormone for enhancing the accumulation of high-value metabolites and stress tolerance in microalgae: applications, mechanisms, and challenges. Bioresour Technol. 2024;393:130093. doi:10.1016/j.biortech.2023.130093. 38000641

[45] Sofy MR, Seleiman MF, Alhammad BA, Alharbi BM, Mohamed HI. Minimizing adverse effects of pb on maize plants by combined treatment with jasmonic, salicylic acids and proline. Agronomy. 2020;10(5):10. doi:10.3390/agronomy10050699.

[46] Metwally A, Finkemeier I, Georgi M, Dietz K-J. Salicylic acid alleviates the cadmium toxicity in barley seedlings. Plant Physiol. 2003;132(1):272–281. doi:10.1104/pp.102.018457. 12746532 PMC166972

[47] Sakouhi L, Kharbech O, Massoud MB, Munemasa S, Murata Y, Chaoui A. Oxalic acid mitigates cadmium toxicity in Cicer arietinum L. Germinating seeds by maintaining the cellular redox homeostasis. J Plant Growth Regul [Internet]. 2022;41(2):697–709. 10.1007/s00344-021-10334-1.

[48] Ma H, Li X, Wei M, Zeng G, Hou S, Li D, Xu H. Elucidation of the mechanisms into effects of organic acids on soil fertility, cadmium speciation and ecotoxicity in contaminated soil. Chemosphere. 2020;239:124706. doi:10.1016/j.chemosphere.2019.124706. 31493754

[49] Montiel-Rozas MM, Madejón E, Madejón P. Effect of heavy metals and organic matter on root exudates (low molecular weight organic acids) of herbaceous species: an assessment in sand and soil conditions under different levels of contamination. Environ Pollut. 2016;216:273–281. doi:10.1016/j.envpol.2016.05.080. 27267743

[50] Ban Z, Niu C, Li L, Gao Y, Liu L, Lu J, Farouk A, Chen C. Exogenous brassinolides and calcium chloride synergically maintain quality attributes of jujube fruit (Ziziphus jujuba Mill.). Postharvest Biol Technol. 2024;216:113039. doi:10.1016/j.postharvbio.2024.113039.

[51] Urmi TA, Islam M, Zumur KN, Abedin M, Haque MM, Siddiqui MH, Murata Y, Hoque Md A. Combined effect of salicylic acid and proline mitigates drought stress in rice (Oryza sativa L.) through the modulation of physiological attributes and antioxidant enzymes. Antioxidants. 2023;12(7):1438. doi:10.3390/antiox12071438. 37507977 PMC10375981

[52] Noctor G, Lelarge-Trouverie C, Mhamdi A. The metabolomics of oxidative stress. Phytochemistry [Internet]. 2015;112:33–53. https://www.sciencedirect.com/science/article/pii/S0031942214003653. 25306398 10.1016/j.phytochem.2014.09.002

[53] Liu J, Wei Z, Li J. Effects of copper on leaf membrane structure and root activity of maize seedling. Bot Stud. 2014;55(1):55. doi:10.1186/s40529-014-0047-5. 28510936 PMC5432969

[54] Reddy AM, Kumar SG, Jyothsnakumari G, Thimmanaik S, Sudhakar C. Lead induced changes in antioxidant metabolism of horsegram (Macrotyloma uniflorum (Lam.) Verdc.) and bengalgram (Cicer arietinum L.). Chemosphere [Internet]. 2005;60(1):97–104. https://www.sciencedirect.com/science/article/pii/S0045653504012007. doi:10.1016/j.chemosphere.2004.11.092. 15910908

[55] Lamhamdi M, Bakrim A, Aarab A, Lafont R, Sayah F. Lead phytotoxicity on wheat (Triticum aestivum L.) seed germination and seedlings growth. C R Biol [Internet]. 2011;334(2):118–126. https://www.sciencedirect.com/science/article/pii/S1631069110002957. 21333942 10.1016/j.crvi.2010.12.006

[56] Yi J, Li H, Zhao Y, Shao M, Zhang H, Liu M. Assessing soil water balance to optimize irrigation schedules of flood-irrigated maize fields with different cultivation histories in the arid region. Agric Water Manag. 2022;265:107543. doi:10.1016/j.agwat.2022.107543.

[57] Soyingbe OS, Ntanzi C, Makhafola TJ, Kappo AP. Ameliorative effect of exogenously applied oxalic acid on nickel heavy metal induced stress in zea mays. Pak J Bot. 2020;52(2):413–418. doi:10.30848/PJB2020-2(14).

[58] Rizwan M, Ali S, Rehman MZ, Javed MR, Bashir A. Lead toxicity in cereals and its management strategies: a critical review. Water Air Soil Pollut [Internet]. 2018;229(6):211. doi:10.1007/s11270-018-3865-3.

[59] Zanganeh R, Jamei R, Rahmani F. Role of salicylic acid and hydrogen sulfide in promoting lead stress tolerance and regulating free amino acid composition in Zea mays L. Acta Physiol Plant [Internet]. 2019;41(6):94. 10.1007/s11738-019-2892-z.

[60] Gill SS, Tuteja N. Reactive oxygen species and antioxidant machinery in abiotic stress tolerance in crop plants. Plant Physiol Biochem [Internet]. 2010;48(12):909–930. https://www.sciencedirect.com/science/article/pii/S0981942810001798. doi:10.1016/j.plaphy.2010.08.016. 20870416

[61] Sidhu GPS, Singh HP, Batish DR, Kohli RK. Alterations in photosynthetic pigments, protein, and carbohydrate metabolism in a wild plant Coronopus didymus L. (Brassicaceae) under lead stress. Acta Physiol Plant. 2017;39(8):39. doi:10.1007/s11738-017-2476-8.

[62] Sharma P, Dubey RS. Lead toxicity in plants. Braz J Plant Physiol. 2005;17(1):35–52. doi:10.1590/S1677-04202005000100004.

[63] Al-Ghzawi AL, Khateeb W, Rjoub A, Al Tawaha AR, Musallam I, Al Sane K. Lead toxicity affects growth and biochemical content in various genotypes of barley (Hordeum vulgare L.). Bulgarian J Agric Sci. 2019;25:55–61.

[64] Song J, Markewitz D, Wu S, Sang Y, Duan C, Cui X. Exogenous oxalic acid and citric acid improve lead (Pb) tolerance of Larix olgensis a Henry seedlings. Forests. 2018;9(9):510. doi:10.3390/f9090510.

[65] Kaur G, Kaur S, Singh HP, Batish DR, Kohli RK, Rishi V. Biochemical adaptations in Zea mays roots to short-term Pb2+ exposure: ROS generation and metabolism. Bull Environ Contam Toxicol. 2015;95(2):246–253. doi:10.1007/s00128-015-1564-y. 26048438

[66] Park JH, Lamb D, Paneerselvam P, Choppala G, Bolan N, Chung J-W. Role of organic amendments on enhanced bioremediation of heavy metal(loid) contaminated soils. J Hazard Mater [Internet]. 2011;185(2–3):549–574. https://www.sciencedirect.com/science/article/pii/S0304389410012434. 20974519 10.1016/j.jhazmat.2010.09.082

[67] Chaudhari S, Upadhyay A, Kulshreshtha S. Influence of organic amendments on soil properties, microflora and plant growth [Internet]. In: Lichtfouse E. editor. Sustainable agriculture reviews. Vol. 52. Cham: Springer International Publishing; 2021. p. 147–191. doi:10.1007/978-3-030-73245-5_5.

[68] Zulfiqar F, Chen J, Finnegan PM, Younis A, Nafees M, Zorrig W, Hamed KB. Application of trehalose and salicylic acid mitigates drought stress in sweet basil and improves plant growth. Plants. 2021;10(6):1078. doi:10.3390/plants10061078. 34072096 PMC8230182

[69] Shan C, Zhang S, Li D, Zhao Y, Tian X, Zhao X, Wu Y, Wei X, Liu R. Effects of exogenous hydrogen sulfide on the ascorbate and glutathione metabolism in wheat seedlings leaves under water stress. Acta Physiol Plant. 2011;33(6):2533–2540. doi:10.1007/s11738-011-0746-4.

[70] Chmielowska-Bak˛ J, Deckert J. Plant recovery after metal stress—a review. Plants2021. 2021;10(3):1–12. doi:10.3390/plants10030450. PMC7997312 33673654

[71] Hayat Q, Hayat S, Alyemeni MN, Ahmad A. Salicylic acid mediated changes in growth, photosynthesis, nitrogen metabolism and antioxidant defense system in Cicer arietinum L. Plant Soil Environ. 2012;58(9):417–423. doi:10.17221/232/2012-PSE.

